# Determinants of anxiety in patients with advanced somatic disease: differences and similarities between patients undergoing renal replacement therapies and patients suffering from cancer

**DOI:** 10.1007/s11255-012-0326-6

**Published:** 2012-11-17

**Authors:** Justyna Janiszewska, Monika Lichodziejewska-Niemierko, Justyna Gołębiewska, Mikołaj Majkowicz, Bolesław Rutkowski

**Affiliations:** 1 Department of Palliative Medicine, Medical University of Gdansk, Dębinki 2, 80-211 Gdańsk, Poland; 2 Department of Nephrology, Transplantology and Internal Medicine, Medical University of Gdansk, Gdańsk, Poland; 3 Department of Research on Quality of Life, Medical University of Gdansk, Gdańsk, Poland

**Keywords:** End-stage renal disease, Renal replacement therapies, End-stage breast cancer anxiety, Coping strategies

## Abstract

**Objective:**

Anxiety is the most frequent emotional reaction to the chronic somatic disease. However, little is known about anxiety and coping strategies in patients with end-stage renal disease (ESRD) undergoing renal replacement therapies (RRTs). The purpose of the study was to assess the intensity and determinants of anxiety in patients treated with different RRTs in comparison with end-stage breast cancer patients and healthy controls.

**Methods:**

The study involved (1) ESRD patients undergoing different RRTs: 32 renal transplant recipients, 31 maintenance haemodialysis and 21 chronic peritoneal dialysis patients, (2) women with end-stage breast cancer (*n* = 25) and (3) healthy persons (*n* = 55). We used State–Trait Anxiety Inventory, Scale of Personal Religiousness, Mental Adjustment to Cancer Scale, Rotterdam Symptom Checklist with reference to medical history. The data thus obtained were analysed using the analysis of variance, the Tukey’s HSD post hoc test and Spearman’s rank correlation coefficient.

**Results:**

Both ESRD and breast cancer patients revealed higher level of anxiety state and trait than healthy controls; however, there was no statistically significant difference found between both findings. There was a tendency towards higher levels of anxiety state in breast cancer patients when compared to ESRD patients undergoing the RRT treatment and for both groups non-constructive coping strategies correlated with the levels of anxiety state. With ESRD patients undergoing RRTs, the intensity of anxiety state did not depend on the mode of treatment but on the correlation between the levels of anxiety and the general quality of their life, psychological condition and social activity.

**Conclusion:**

In patients with advanced somatic disease (ESRD and end-stage breast cancer), non-constructive strategies of coping with the disease require further evaluation and possibly psychological support.

## Introduction

End-stage renal disease (ESRD) has become a major increasingly prevalent health issue. Patients’ permanent loss of renal function requiring a chronic renal replacement therapy (RRT) leads to dramatic life changes. RRTs prolong the lives of ESRD patients but at the same time bring them an enormous burden. A broad range of physical symptoms, activity limitations, altered body image, fatigue and various treatment-related restrictions are stressors precipitating the need for coping efforts and possibly leading to negative outcomes such as depression and anxiety. Therefore, psychological disorders are frequently encountered in such cases [1, 2].

Anxiety is an unpleasant emotion affecting patients with advanced somatic disease, and there are various ways of coping with it. Most scientists who deal with the issue of emotional reactions to somatic disease point out that it is anxiety which is the most common emotional response to somatic diseases [3–6]. Anxiety is a response to either a threatening situation, a risk of losing an important value or a lack of stability, that is, the feeling of insecurity connected with finding oneself in a new unfamiliar situation [5]. We can assess anxiety as a fairly stable anxiety-related trait combined with personality features and a variable anxiety-induced state, which depends on external factors. Getting ill involves the risk of a patient’s permanent loss of health, development of disability or even death. At the same time, the disease changes the level of one’s self-esteem and one’s previous ways of dealing with problems. Thus, the phenomenon of anxiety occurs at the very onset of disease symptoms during diagnostic and treatment stages, which follow, especially if hospitalization is necessary [5].

It has been frequently observed that there is a clear association between the patient’s religiousness and spirituality, on the one hand, and clinical outcomes on the other. One’s spirituality and religious involvement may play the key role in developing coping mechanisms, which—as has been verified—both improve one’s quality of life (QoL) and reduce one’s perception of the burden of illness and of its depressive effects [7, 8]. Spinale et al. [9] showed that highly developed spirituality resulted in patients’ improved chances of survival with regard to the haemodialysis population, but religion as a coping mechanism did not. In end-stage cancer patients, religiousness turned out to be an effective tool for coping with anxiety. Because little is known about anxiety and the coping strategies in patients undergoing renal replacement therapies, it is also not known if religiousness is a determinant of anxiety in ESRD population.

## Objectives

The detailed aims of the study were

- To establish the intensity of anxiety trait and anxiety state in study groups.
- To find the relationship between physical symptoms, psychological symptoms, social activity and general quality of life and level of anxiety patients undergoing different RRTs (haemodialysis, peritoneal dialysis, renal transplant recipients) and cancer patients.
- To assess the association between anxiety state and coping strategies in patients.
- To find out if religiousness has an impact on anxiety levels in the studied groups.

## Materials and methods

The study involved 164 subjects (85 women and 79 men), aged between 22 and 80 (average age: 49.17) who comprised three study groups: the first group (I) consisted of patients undergoing different modes of RRT (*n* = 84) divided into three groups according to method of treatment: 32 renal transplant patients (RTx), 31 maintenance haemodialysis (HD) and 21 chronic peritoneal dialysis (PD) patients; the second group (II) included terminal breast cancer patients (*n* = 25). These patients found themselves relapsing further into disease and as a result experienced a significant limitation of their professional and social activities; the third group (III) comprised healthy persons (*n* = 55). The following instruments were used to conduct the study:

1. State–Trait Anxiety Inventory (STAI) in an authorized translation by J. Strelau, M. Tyskarczyk and K. Wrześniewski [10]. It is an adaptation of the American version of the State–Trate Anxiety Inventory developed by Spielberger et al. [11]. This instrument enables one to differentiate and to measure independently two different types of anxiety, namely anxiety understood both as the current state of an individual (“state anxiety”) and as a relatively stable trait of this individual’s personality (“trait anxiety”). Both scales contain 20 short statements assessing the patient’s subjective feelings. The received results are compared with suitable centile norms. Results under 30 centile show the low level of anxiety, between 30 and 60 centiles reflect the normal level of anxiety and more than 60 centile indicate the high level of anxiety. This inventory is an instrument that lives up to the requirements of modern psychometry. It has high reliability and accuracy indices, respectively. Estimates of reliability (Cronbach’s alpha) of the Polish version of the Scale were satisfactory for two scales Anxiety—State (alpha = .90) and Anxiety—Trait (alpha = .88) [10].
2. Scale of Personal Religiousness (SPR) [12]. This scale was created by Jaworski. It measures religious attitudes and enables one to differentiate between personal religiousness and the apersonal one. Personal religiousness means deeply rooted faith in God that is expressed through religious practices, spiritual development and being a member of a church community following religious rules in daily life. Apersonal religiousness is the opposite of the previous attitude: it reduces faith to tradition, ceremonies and superficial acts of worship. The maximum result—210 points—indicates the personal character of religiousness. The lowest score—30 points—is the evidence of apersonal religiousness. There is a continuum of varied religiousness concerning assessment of both personal and apersonal attitudes towards religion. This scale was created in Polish. The reliability of the scale (Cronbach’s alpha) was proven of retesting method where satisfactory: for particular questions (alpha = .69–.86) [12].
3. Rotterdam Symptom Checklist (RSCL) [13]. The authors of the Polish version of the questionnaire are Majkowicz and De Walden-Gałuszko [13]. It is an adaptation of the Scale developed by JCM de Heas, M. Olschewski and P. Fayers et al. This checklist assesses the quality of life in cancer patients throughout self-description. The original version of the RSCL covers four areas: (a) physical (somatic) symptoms, (b) psychological problems, (c) activity level and (d) the overall quality of life [14]. On the basis of the results and conclusions drawn from the overall quality of life study in the general population, five statements were added regarding the question of social activity. Estimates of reliability (Cronbach’s alpha) of the Polish version of this questionnaire were highly: for physical symptoms scale (alpha = .86), psychological symptoms scale (alpha = .83) and activity scale (alpha = .87) [13].
4. The Mini-Mental Adjustment to Cancer Scale (Mini-MAC) [16] measuring the following coping strategies: (a) fighting spirit attitude—facing up to the challenge and fighting the disease, (b) anxiety preoccupation—continuous fear and thinking about the disease, (c) helplessness–hopelessness—feeling resigned to disease, (d) cognitive avoidance—adopting a new, re-considered approach to disease and developing a more appreciative attitude to life as a value itself (e) fatalism—stoicism, acceptance of disease as the will Providence.

The author of the Polish version of the Scale is Z. Juczyński. It is an adaptation of the American version of the test developed by Watson et al. [15].

The Scale included 29 statements. The results must be estimated according to each of four strategies separately. There are seven statements in each strategy. The range of possible results should be contained within the limit of 7–28 points. The higher the score the larger the intensification becomes of the characteristic modes of behaviour for a specified way of coping with disease. The scores thus obtained should be converted into standardized units—by means of so-called stens (i.e. standardized ten-point scales). Results from 1 to 4 need to be treated as low while those from 7 to 10 should be regarded as high. Results in limits 5 and 6 be classified as mean estimates of reliability (Cronbach’s alpha) of the Polish version of the Scale were highly: for helplessness–hopelessness strategy (alpha = .92), fighting spirit attitude (alpha = .90), anxiety preoccupation strategy (alpha = .89), cognitive avoidance strategy (alpha = .87), fatalism (alpha = .64) [16].

5. Medical records served as the source of data with regard to methods of treatment, comorbidities, social and demographic details and selected laboratory indices (Kt/V for dialysis adequacy in HD and PD patients and eGFR for kidney allograft function), haematocrit levels.
6. Charlson Comorbidity Index (CCI) [17] was used as a single aggregate measure of a patient’s comorbid conditions. CCI scores were calculated according to age modification as proposed by Charlson et al. [17].

## Statistical methods

Statistical analysis was conducted using the Statistica 9 program. Spearman’s rank correlation coefficient was used for analysing the correlation level between variables. The influence of independent variables on the dependent variables was determined by means of variance analysis (ANOVA). Differences among subgroups were assessed using the Tukey’s test. A *p* value <.05 was considered statistically significant. Data are shown as mean ± standard error.

## Results

Basic characteristics of studied groups are presented in Table 1. Patients with end-stage breast cancer were women. They displayed no symptoms of comorbidity. Among patients undergoing RRTs, RTx recipients were younger than both HD and PD patients; standard mean ages of dialysis patients were comparable. Healthy controls were the youngest of all the studied groups. CCI scores were the lowest for RTX patients and the highest for HD patients. These findings bore statistical significance.

**Table 1:** Characteristics of studied group

Group	Age (years)	*p*					Sex		Time of treatment (years)	CCI	Kt/V eGFR	Hct (%)
	*x* ± SD	I			II	III	Women	Men	*x* ± SD	*x* ± SD	*x* ± SD	*x* ± SD
		RTx	HD	PD			*N*	*N*				
I—RRT	53.56 ± 13.94	–			.06	.02*	33	51	3.54 ± 1.47	–	–	–
RTx	44.37 ± 12.87	–	.00*	.00	–	**–**	13	18	4.45 ± 2.26	4.06 ± 1.18	45.31 ± 12.36	34.4 ± 5.6
HD	59.71 ± 12.22	.00*	–	.94	–	**–**	9	22	2.34 ± 2.30	6.28 ± 1.68	1.57 ± .15	34.9 ± 4.7
PD	58.48 ± 10.30	.00*	.94	–	–	**–**	22	10	3.94 ± 3.43	5.67 ± 2.07	2.35 ± .37	35.3 ± 2.5
II—breast cancer	62.3 ± 12.21		.06		–	.00*	25	–	5.5 ± 2.52	–	–	36.9 ± .8
III—healthy controls	44.8 ± 9.23		.02*		.00*	–	27	28	–	–	–	–

*p* coefficient of statistical significance

* The marked values are statistically significant, with *p* < .05

Statistical analysis confirmed a significant positive relationship between trait anxiety and state anxiety. Analysis of the results showed that patients with advanced somatic illness (ESRD, breast cancer) revealed higher levels of anxiety state and trait in comparison with healthy controls; however, the results did not reach statistical significance. There was a tendency towards higher level of anxiety state in breast cancer patients as compared to ERSD patients undergoing RRT; HD, PD and RTx patients had similar intensity of anxiety state too. Results are presented in Table 2.

**Table 2:** The level of anxiety state and trait in studied groups (data shown in centiles)

Anxiety		Group					
		I—RRT patients				II—breast cancer patients	III—healthy controls
		RTx	HD	PD	Total		
State	*x*	51.45	47.53	49.19	49.44	58.20	47.05
	SD	10.91	10.85	11.29	10.59	13.26	14.08
Trait	*x*	50.19	51.92	49.39	50.36	49.63	45.68
	SD	9.35	9.96	11.13	10.31	9.35	8.32

Tukey’s HSD Post hoc test confirmed no significant relationship (*p* > .05) between level of anxiety and gender in ESRD patients and healthy control groups.

The coping strategies as helplessness–hopelessness, anxiety preoccupation correlated with level of anxiety state in ESRD patients undergoing RRT. The coping strategies as fatalism and anxiety preoccupation correlated with levels of anxiety state in women affected by breast cancer.

In contrast to breast cancer patients, in ESRD patients religiousness had no effect on their anxiety state. Spearman’s rank correlation coefficient values for the variables state anxiety, religiousness and patient ways of coping are presented in Table 3.

**Table 3:** The correlation between anxiety state and coping strategies in studied groups

Variables	Group									
	ESRD patients								Breast cancer patients	
	RTx		HD		PD		Total			
	*r*	*p*	*r*	*p*	*r*	*p*	*r*	*p*	*r*	*p*
Religiousness	−.18	.33	.14	.45	−.09	.71	−.03	.79	−.74	.000*
Helpless–hopeless	.57	.001*	.64	.000*	.61	.004*	.37	.001*	.82	.03*
Fatalism	−.15	.40	.10	.60	.22	.34	−.09	.40	.79	.000*
Anxious preoccupation	.66	.000*	.66	.000*	.74	.004*	.50	.000*	.71	.000*
Cognitive avoidance	.15	.42	−.048	.83	.18	.46	.048*	.71	.81	.04*
Fighting spirit	.17	.36	−.10	.59	−.14	.56	−.17	.14	.80	.02*

*r* the Spearman’s rank correlation coefficient

*p* coefficient of statistical significance

* The marked values are statistically significant, with *p* < .05

QoL was assessed with the help of RSCL. There were more physical symptoms in breast cancer patients in comparison with dialysis and renal transplant patients. On the other hand, cancer patients evaluated higher their overall QoL when compared with ESRD patients. In RTx patients, general QoL was significantly better than in HD patients. There was a difference of borderline significance in the general QoL evaluation between RTx and PD patients (*p* = .054). General QoL did not differ between HD and PD patients. Psychological symptoms and activity level did not differ between all groups (cancer and RRT patients). RTx patients suffered from as many physical and psychological symptoms as dialysis patients, yet their self-sufficiency was rated significantly higher than in HD patients. In ESRD group, there was a significant positive correlation between physical symptoms, activity level and overall QoL and CCI scores. RTx recipients with better kidney allograft function assessed by eGFR experienced fewer physical symptoms. There was no such interrelationship with regard to dialysis adequacy assessed by Kt/V in both HD and PD patients (data not shown). The haematocrit (Hct) levels were measured. These results did not differ between studied groups.

Table 4 shows the correlation between anxiety state and various domains of QoL in ESRD and cancer patients. There was a correlation between level of anxiety and psychological symptoms and social activity in RRT patients. In RTx patients, level of anxiety was also negatively correlated with general QoL, whereas in HD patients it did not correspond to the level of social activity. There was no correlation between somatic symptoms and level of anxiety in any of ESRD patients, regardless of the mode of RRT. In contrast to RTx, HD and PD patients, in breast cancer patients, the intensity of anxiety corresponded to the results in all domains of RSCL.

**Table 4:** The correlation between physical symptoms, psychological symptoms, social activity and general QoL and level of anxiety in ESRD and cancer patients

Scale of RSCL	Group									
	RRT patients								Breast cancer patients	
	RTx		HD		PD		Total		*r*	*p*
	*r*	*p*	*r*	*p*	*r*	*p*	*r*	*p*		
Physical symptoms	.30	.10	.00*	.96	.32	.16	.15	.17	.44	.02*
Psychological symptoms	.66	.00*	.57	.00*	.61	.00*	.38	.00*	.69	.00*
Activity level	.55	.00*	.08	.67	.46	.04*	.30*	.00*	.19	.01*
General QoL	.54	.00*	.08	.67	.19	.44	.21	.06	.74	.03*

*r* the Spearman’s rank correlation coefficient

*p* coefficient of statistical significance

* The marked values are statistically significant, with *p* < .05

There was no significant relationship between level of anxiety and time of treatment in the studied groups.

## Discussion

The findings of the research being presented here have confirmed that patients with higher levels of trait anxiety who suffer from advanced somatic disease reveal more intensive state anxiety. It seems to be obvious that people with anxiety as a feature of character will become more anxious when they have to cope with this disease. We observed that patients with advanced somatic illness (ESRD, breast cancer) revealed higher level of anxiety state and trait in comparison with healthy controls; however, the results did not reach statistical significance. These results suggest that anxiety is the frequent emotional reaction to the chronic somatic disease and can be compared with the obtained from previous studies conducted on patients undergoing haemodialysis treatment [18–20].

Anxiety trait is understood as a relatively stable trait of an individual’s personality. This may suggest that chronic somatic disease can cause a change of personality.

Patients with breast cancer revealed higher levels of anxiety than other studied patients including ESRD patients. In a study by Majkowicz et al. [21], cancer patients expressed significantly higher levels of depression and anxiety while HD patients manifested higher levels of aggression. Moreover, it is typical of end-stage cancer to make existing symptoms become periodically aggravated and cause new ones to appear. New symptoms will tend to arouse anxiety [22]. The fact that cancer patients were all women could be important for the level of anxiety. Some researchers point to gender dimension as one of the factors influencing on anxiety level [23, 24]. A study carried out by Spencer et al. [25] showed that female patients, more physically impaired and younger advanced cancer patients are more likely to meet criteria for an anxiety disorder.

As expected, terminal cancer patients rated their somatic condition worst of all study groups. It is surprising that cancer patients scored overall QoL better when compared to dialysis and renal transplant patients. The possible explanation is that renal patients tend to be affected by medical therapy more heavily. In many countries, this is the reason for the withdrawal of aggressive treatment for dialysis patients [26].

Renal transplantation is the ideal choice of treatment for ESRD. Not only does it improve one’s chances of survival, but it is believed to be the least stressful of RRTs since it brings patients fewer life style restrictions and improves their physical and psycho-social well-being. Therefore, successful transplantation should mitigate high level of negative emotions observed in dialysis patients. Among studies comparing the condition of mental health between haemoperitoneal dialysis and transplant patients, some have shown an improvement in anxiety after renal transplantation [27, 28], while others have revealed no sign of superiority of any of the RRT modes [29].

In our study, HD, PD and RTx patients had similar intensity of anxiety state, while their general evaluation of QoL differed with RTx recipients experiencing best QoL. This was surprising as RTx patients were younger and manifested fewer instances of comorbidity than dialysis patients. We would have expected these patients to express lower levels of negative emotions than HD or PD patients. Our findings are especially surprising as most of the previous studies showed a favourable effect of renal transplantation on the improvement in anxiety state [27, 28]. We would well have expected PD patients to have a better QoL than their HD counterparts, even comparable to healthy individuals, for example, because this method of RRT allows increased autonomy [20, 21]. On the other hand, in a study by Sayin et al. [29], QoL was similar to that of HD, PD and RTx patients. However, anxiety levels influenced QoL in the case of HD and PD patients alone—RTx recipients remained unaffected. This is consistent with our observation that while all patients undergoing RRTs maintained similar anxiety state levels, RTx patients scored best overall QoL. We can thus hypothesize that RTx patients, who have the highest functional capacity and QoL, become anxious about a possible loss of these values. Although RTx patients suffered from as many physical and psychological symptoms as dialysis patients did, their self-sufficiency, constituting a highly appreciated quality, was enough to boost their QoL.

Intrinsic religiousness was the most effective factor diminishing anxiety in advanced breast cancer patients as opposed to dialysis and transplant ones. Women with intrinsic religiousness show the lower intensity of anxiety; on the other hand, patients with extrinsic religiousness display a higher level of anxiety. Some researchers point to the transcendental dimension as one of the mechanisms for coping with the fear of death [30–33]. Alvarado et al. [34] showed that people with low levels of the fear of death strongly believe in afterlife. Moreover, a study by Phelps et al. [35] showed that religious coping in advanced cancer patients is associated with receipt of intensive life-prolonging medical care near death defined as receipt of mechanical ventilation or cardiopulmonary resuscitation in the last week of life.

The analysis of ways of coping with disease revealed that the strategies as helplessness–hopelessness, anxiety preoccupation correlated with level of anxiety state in patients with ESRD undergoing RRT and the strategies as fatalism and anxiety preoccupation correlated with level of anxiety state in women with breast cancer. The coping strategies were assigned to either of two categories: constructive coping strategies (fighting spirit, cognitive avoidance) and non-constructive coping strategies (helpless–hopeless, anxious preoccupation, fatalism). We observed that non-constructive ways of coping correlated with higher level of anxiety in studied groups. Our study on 155 women with breast cancer at different disease stages has shown that patients preferring constructive coping strategies revealed lower levels of anxiety when compared to patients manifesting non-constructive forms of coping [36]. Our results concerning patients who receive RRTs are consistent with the report of Takaki et al. [37], who have shown that emotion-oriented coping such as anxiety preoccupation correlated positively with level of anxiety in over 400 HD patients.

In ESRD patients’ correlation between level of anxiety and general quality of life, psychological state and social activity depended on mode of treatment. There was no correlation between somatic symptoms and levels of anxiety in the case of dialysis and renal transplant patients. The breast cancer patients’ intensity of anxiety correlated with the results in all domains of RSCL. In the phase of recurring cancer, the somatic condition has the most significant influence on the levels of state anxiety. Somatic signs at this stage of the disease worsen patients’ health condition and bring about their feeling of threat.

Study limitations include the potential for self-report response bias and sampling bias because of convenience sampling. We also decided to use tools originally designed for cancer patients that so far have not been verified with ESRD patients. However, this was the only possible way to make comparisons between patients suffering from advanced stages of renal diseases and cancer.

In addition, we cannot exclude the idea that a tendency towards higher levels of anxiety state among cancer—affected patients may be associated with their gender. Despite these limitations, we believe that our study is of significant importance as to our knowledge this is the first study to compare anxiety and coping strategies along with religiousness in patients undergoing three different treatment strategies in ESRD. Moreover, our findings thus obtained are further juxtaposed with those from research done into healthy individuals, on the one hand, and cancer patients on the other.

## Conclusions

Patients with a high level of trait anxiety show higher intensity of state anxiety during advanced somatic disease. ESRD patients undergoing RRT present higher level of anxiety than healthy controls and lower than breast cancer patients. The intensity of anxiety is similar irrespectively of the mode of renal replacement therapy (RTx, HD and PD). An elevated level of anxiety trait in patients with advanced disease suggests that the presence of chronic somatic illness modifies this personality feature. The phenomenon of intrinsic religiousness works as a factor most efficiently diminishing the levels of anxiety in advanced breast cancer patients as opposed to dialysis and transplant patients. In patients with advanced somatic disease (ESRD patients and women with breast cancer), non-constructive strategies of coping with the disease require further evaluation and possibly more psychological support.

## Abbreviations

CCI: Charlson comorbidity index
eGFR: Estimated glomerular filtration rate
ESRD: End-stage renal disease
Hct: Haematocrit
HD: Haemodialysis
Kt/V: Urea clearance over dialysis time/volume of distribution of urea
Mini-MAC: The Mini-Mental Adjustment to Cancer Scale
*p*: Coefficient of statistical significance
PD: Peritoneal dialysis
*r*: The Spearman’s rank correlation coefficient
RRT: Renal replacement therapy
RSCL: The Rotterdam Symptom Checklist
RTx: Renal transplant patients
SPR: The Scale of Personal Religiousness
STAI: The State–Trait Anxiety Inventory
QoL: Quality of life
